# Molecular identification and phylogenetic analysis of chronic bee paralysis virus in Iran 

**Published:** 2017-12-15

**Authors:** Mohammadreza Ghorani, Arash Ghalyanchi Langeroudi, Omid Madadgar, Mohammadreza Rezapanah, Sedigheh Nabian, Reza Khaltabadi Farahani, Hossein Maghsoudloo, Mohammad Forsi, Hamed Abdollahi, Hesameddin Akbarein

**Affiliations:** 1 *Department of Microbiology and Immunology, Faculty of Veterinary Medicine, University of Tehran, Tehran, Iran;*; 2 * Center of Excellence for Organic Agriculture and Department of Biological Control, Iranian Research Institute of Plant Protection, Agricultural Research, Education and Extension Organization (AREEO), Tehran, Iran; *; 3 * Department of Parasitology, Faculty of Veterinary Medicine, University of Tehran, Tehran, Iran; *; 4 * Iranian Veterinary Organization, Tehran, Iran; *; 5 * Department of Food Hygiene and Quality Control, Faculty of Veterinary Medicine, University of Tehran, Tehran, Iran*

**Keywords:** Chronic bee paralysis virus, Honeybee, Iran, Phylogenetic analysis, RT-PCR

## Abstract

Chronic bee paralysis virus (CBPV) is an unclassified polymorphic single-stranded RNA virus. Among the viruses infecting honeybees, CBPV is known to induce significant losses in honeybee colonies. In this study, a total number of eighty-nine suspected apiaries from four regions of Iran (including Mazandaran, Khorasan Razavi, Hormozgan, and Kurdistan) were sampled and submitted for molecular identification. Three positive samples were detected by RT-PCR. All positive samples were confirmed by sequencing. The phylogenetic tree which displays the molecular relationship between the viruses of different Iranian geographic regions and references isolates was constructed. The Iranian isolates formed two distinct phylogenetic groups (Group 1 and Group 2). The IR-CPV-GMG-1, IR-CPV-GMG-2, IR-CPV-GMG-4, and IR-CPV-GMG-6 formed Group 1 and IR-CPV-GMG-3, IR-CPV-GMG-5, and IR-CPV-GMG-7 were in Group 2 as a distinct group. Iranian isolates in group 1 were similar to European and East Asian CBPVs. This research was the first phylogenetic analysis of CBPV in Iran. Further researches are needed to study the other aspects of this virus-like genetic characteristics and pathogenesis in Iran.

## Introduction

Chronic bee paralysis formally called paralysis, is an infectious and contagious disease affecting adult honeybees.^1^ Although the symptoms of paralysis were probably recognized more than two thousand years ago by Aristotle, who described hairless black bees that he called them ‘‘thieves”, the causative agent was not confirmed until 1963 when Bailey and colleagues isolated and characterized chronic bee paralysis virus (CBPV).^1^ Together with the acute bee paralysis virus (ABPV), these two viruses were the first two honeybee viruses to be isolated. No links were established between CBPV dissemination or paralysis outbreaks and the important mite parasite *varroa* *destructor*, which is associated with numerous other honeybee viruses.^2^ CBPV is the only common viral disease of adult bees whose symptoms include both visible behaviour modifications and physiological modifications. Paralysis symptoms including trembling and clusters of flightless bees crawling at the hive entrance are previously described.^1^

CBPV is responsible for chronic paralysis, an infectious and contagious disease of adult honeybees (*Apis mellifera *L.). This disease presents well-defined symptoms including abnormal trembling of the wings and bodies of diseased bees which are unable to fly but often crawl on the ground.^3^ Some individuals become almost hairless, and therefore darker in appearance, and suffer nibbling attacks by the healthy bees in their colony. Diseased bees die within a few days. CBPV can persist as an inapparent infection but may multiply to high levels in honeybees^4^ and cause significant losses in colonies.^5^ Nutritional deficiency, severe winters or bad weather conditions in summer may favor disease outbreaks.^5^^,^^6^ The distribution of this virus is worldwide,^5^ most likely resulting from the intensive commercial exchange of honeybees.

Current diagnosis of the clinical disease is based on RT-PCR tests which allow the detection of CBPV even in asymptomatic colonies.^7^^,^^8^ Using RT-PCR based assays, the virus infections in honeybees can be detected and quantified in a rapid and accurate manner.^9^^,^^10^ Our objective was to evaluate the presence of CBPV in honeybees' colonies in Iran.

The CBPV was first isolated from diseased honeybees in 1963.^11^ The etiological agent of this paralysis is CBPV, an unclassified polymorphic (particles 20 × 30 to 60 nm) single-stranded RNA virus.^12^

The CBPV genome was originally described as containing five single-stranded RNA fragments: Two major RNAs, RNA 1 and RNA 2 of about 4200 and 2800 nucleotides, respectively, and three minor RNAs, RNA 3a, 3b and 3c, each of about 1100 nucleotides.^13^ Fingerprint analysis showed that (i) these RNAs had different sequences, (ii) they seemed identical to the minor CBPV RNAs species, 3a, 3b and3c and (iii) they presented sequence similarities with CBPV RNA 2.^13^ The morphology of the CBPV particles and the multipartite organization of the RNA genome are exceptional, as most honeybees viruses are picorna-like viruses in the *Iflavirus *and *Cripavirus *genera with symmetric particles and positive single-stranded RNA genomes.^14^ The CBPV is currently classified as an RNA virus but is not included in any family or genus. Recent advances in molecular technology have greatly expanded our ability to detect and elucidate the molecular events associated with virus infections and pathogenesis.^15^^,^^16^

The aims of this study were to use RT-PCR for the sensitive direct detection of CBPV in clinical samples, to reveal and compare the nucleotide sequences of the CBPV nonstructural polyprotein RdRp gene region of different Iranian isolates, and to assess the genetic relationship between CBPV strains of various geographic origins. Here, we described the first phylogenetic analysis of CBPV in Iran. The phylogenetic tree was constructed for Iranian isolates of CBPVs.

## Materials and Methods

**Fig. 1:**
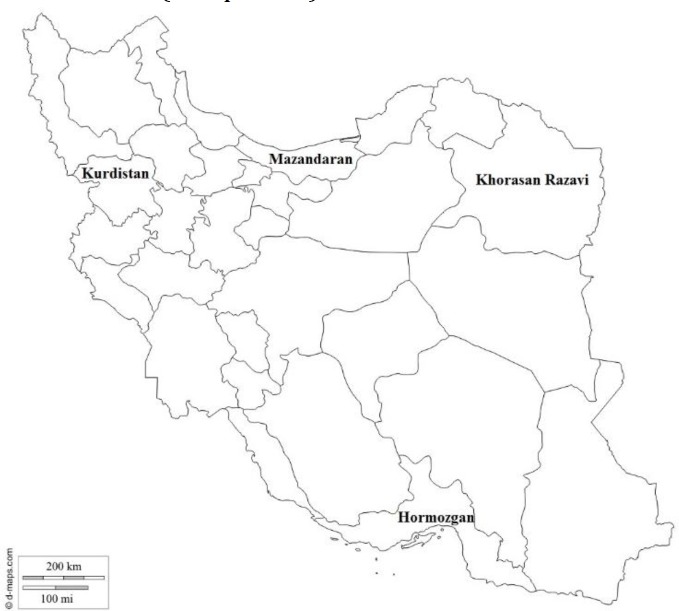
The distribution pattern of four provinces of Iran where honeybee samples were collected. The North: Mazandaran, The South: Hormozgan, The East: Khorasan Razavi, and The West: Kurdistan.

**Study area and sample collection. **We chose four different geographical provinces of Iran (The North: Mazandaran, The South: Hormozgan, The East: Khorasan Razavi, and The West: Kurdistan) (Fig. 1). Geographical feature of these regions is shown in Table 1. Beekeeping industries are developed in these provinces. Beekeepers submitted bee samples from colonies suffering from symptoms of depopulation, sudden collapse, paralysis or dark colour. The samples from Mazandaran (23 apiaries), Hormozgan (20 apiaries), Khorasan Razavi (23 apiaries), and Kurdistan (23 apiaries) were collected. 

**Table 1:** The geographical features of four Iranian provinces using for sampling.

**Province **	**Geographical location**	**Area**
**Mazandaran**	36.5656°N 53.0588°E	23,833 km^2^
**Hormozgan**	27.1884°N 56.2768°E	70,697 km^2^
**Khorasan Razavi**	36.2980°N 59.6057°E	118,884 km^2^
**Kurdistan**	35.3113°N 46.9960°E	29,137 km^2^

Altogether, samples from 89 apiaries were randomly collected and submitted for virus screening. From each apiary, 250 adult dead worker bees were sent to the laboratory. All bee samples from each apiary were pulled. Bee samples were collected from October 2015 to September 2016 and referred to the microbiology and immunology lab of the faculty of Veterinary Medicine, University of Tehran. The honeybee specimens were stored at –20 ˚C until processed.

**RNA extraction and cDNA synthesis. **The bees were homogenized in ceramic mortars with sterile diethylpyro-carbonate treated water (Sinaclone, Tehran, Iran). The homogenates were centrifuged at 20,000 *g *for 1 min, and 140 μL of supernatant was used for RNA extraction.^17^ Viral RNA was isolated using a silica-based CinnaPure RNA extraction kit (Sinaclone) according to manufacture instructions. For cDNA synthesis, 1 μL (0.20 μg) of random hexamer primer (SinaClone) was added to 5 μL of extracted RNA, and the mixture was heated at 65 ˚C for 5 minutes. Fourteen μL of cDNA master mix containing 7.25 μL of diethylpyrocarbonate treated water (Sina-Clone), 2 μL of dNTP mix (SinaClone), 0.25 μL of RiboLock RNase Inhibitor (Thermo Fisher Scientific Inc., Waltham, USA), 0.50 μL of Revert Aid Reverse Transcriptase (Thermo Fisher Scientific Inc.), and 4 μL of 5X RT reaction buffer was added to each tube, resulting in a final volume of 20 μL. Then, the mixture was incubated at 25 ˚C for 5 min, 42 ˚C for 60 min, 95 ˚C for 5 min, and 4 ˚C for 1 min. The cDNA was stored at –20 ˚C until use. 

**RT-PCR for CBPV detection. **The RT- PCR for CBPV detection based on the nonstructural polyprotein RdRp gene was chosen for this study. For detecting CBPV infection in the honeybee, a forward primer (5′**-**AGTTGTC ATGGTTAACAGGATACGAG**-**3′), and a reverse primer (5′**-**TCTAATCTTAGCACGAAAGCCGAG**-**3′) that lead to a fragment of 455 bp were used according to a previous work by Ribière* et al*.^8,17^ The RT-PCR was carried out using these primers. The PCR condition for amplification was 95 ˚C for 5 min, 35 cycles of 95 ˚C for 30 sec, 50 ˚C for 30 sec, and 72 ˚C for 30 sec, followed by 72 ˚C for 10 min. The products were detected by gel electrophoresis, have been sent for sequencing for confirmation. The RT-PCR products were sequenced by Bioneer Corporation (Daejeon, South Korea).

**Bioinformatics and phylogenetic analysis. **An AccuPrep^®^ PCR Purification Kit (Bioneer) was used for the purification of the PCR products. Sequencing was with the primers (both directions) that were used after PCR (Bioneer). A phylogenetic tree was constructed by the neighbour-joining method, using MEGA software (version 7.0.21; BioDesign Institute, Tempe, USA).^18^ The nucleotide sequences of a nonstructural polyprotein RdRp gene were compared with several nonstructural polyprotein RdRp sequences from GenBank. The CBPV sequences were aligned and compared with reference strains. The obtained sequence was submitted to the NCBI GenBank database. The country of origin, strain name, isolation year and accession number of CBPV strains are shown in Table 2. 

**Table 2:** Data for reference CBPV isolates used in this study.

**Isolate name**	**Country**	**Year**	**Accession number**
**LN-SY**	China	2015	KU950353
**B-441**	France	2007	EU122231
**3657**	China	2015	KX168412
**AT34**	Austria	2004	FJ345307
**Serbia C1**	Serbia	2013	KM001900
**Japan-2**	Japan	2011	AB682796
**198**	France	2007	FJ345313
**23**	Belgium	2006	FJ345309

## Results

**Fig. 2:**
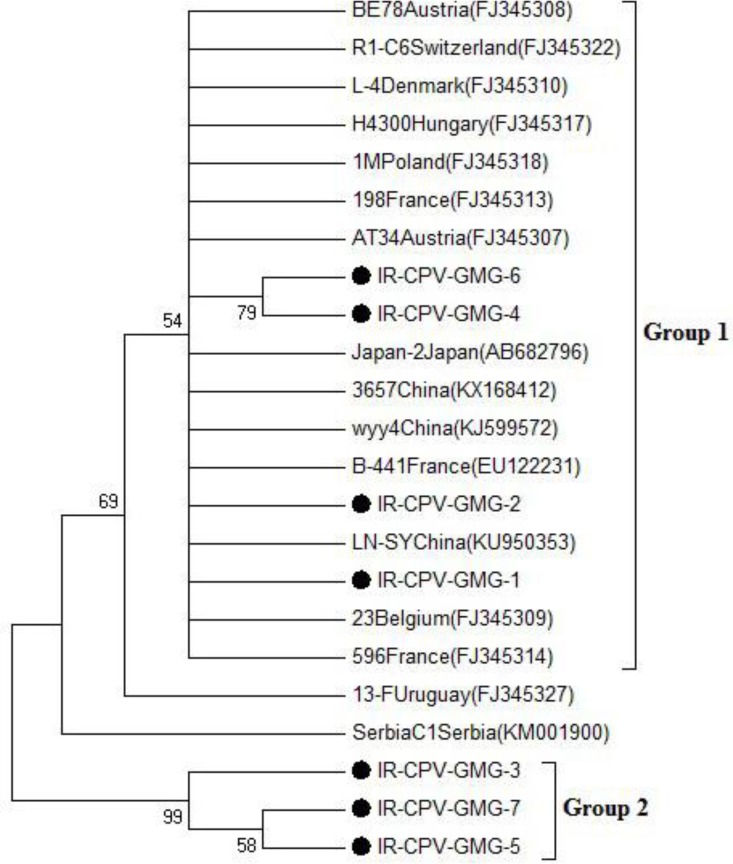
Phylogenetic trees derived from the nonstructural polyprotein RdRp. Phylogenetic tree indicating the genetic relationship between Iranian CBPV (IR-CPV-GMG-1 to IR-CPV-GMG-7) samples based on nonstructural polyprotein RdRp gene according to reference strains.

Phylogenetic analysis based on nonstructural polyprotein RdRp nucleotide sequences showed that Iranian CBPV isolates fell into two groups (Fig. 2). 

Out of the 89 apiaries examined, 3 (3.37%) were infected with CBPV (Khorasan Razavi: 2, Kurdistan: 0, Hormozgan: 0, Mazandaran: 1). All positive samples were confirmed by sequencing (Table 3).

**Table 3:** Percent identity of partial nucleotide sequences of the nonstructural polyprotein RdRp genes of some Iranian CBPVs to those of CBPV reference strains.

	**IR-CPV-GMG-6**	**IR-CPV-GMG-4**	**IR-CPV-GMG-2**	**IR-CPV-GMG-1**	**Serbia C1**	**23**	**LN-SY**	**B-441**	**3657**	**Japan-2**	**AT34**	**198**
**198**	92.3	93.2	97.5	55.7	93.7	97.9	97.8	96.3	97.1	97.1	99.3	100
**AT34**	92.3	94	97.5	55.5	93.6	97.9	97.8	96.3	97.1	97.1	100	99.3
**Japan-2**	91.4	92.4	98.9	57.4	93.7	96.4	99.3	97.8	98.6	100	97.1	97.1
**3657**	90.6	92.3	99.6	57.5	94.4	96.4	99.3	97.8	100	98.6	97.1	97.1
**B-441**	90	91.5	98.2	56.4	92.8	95.6	98.6	100	97.8	97.8	96.3	96.3
**LN-SY**	91.4	92.3	99.6	59.4	93.7	97.1	100	98.6	99.3	99.3	97.8	97.8
**23**	92.3	93.6	96.7	56.8	95.2	100	97.1	95.6	96.4	96.4	97.9	97.9
**Serbia C1**	90.6	92.3	94.1	45.2	100	95.2	93.7	92.8	94.4	93.7	93.6	93.7
**IR-CPV-GMG-1**	40.13	37.38	58.45	100	45.18	56.75	59.36	56.44	57.51	57.38	55.5	55.65
**IR-CPV-GMG-2**	90.99	91.93	100	58.45	94.05	96.73	99.64	98.21	99.64	98.94	97.47	97.48
**IR-CPV-GMG-4**	93.98	100	91.93	37.38	92.34	93.58	92.34	91.46	92.34	92.35	93.99	93.2
**IR-CPV-GMG-6**	100	93.98	90.99	40.13	90.58	92.29	91.41	90.04	90.57	91.42	92.28	92.29

## Discussion

Honeybees play an important role in pollinating, thus provide billions of dollars and added value in agriculture. In Iran, due to the large areas and different weather conditions, beekeeping industry has a potential feasibility. According to the national data, Iran is located in the Ninth world as a number of colonies and the twelfth world as a producing honey.^19^

In France, CBPV has been detected by serology in extracts of dead adult *Apis* *mellifera*, thus confirming that overt disease can be present at any time of the year.^20^ Tentcheva *et al*. also reported the sporadic detection of CBPV over the year in adult bees sampled from 360 apparently healthy colonies in different regions of France.^21^ CBPV was found in adult bees in 28.00% of the 36 surveyed apiaries.^21^ The virus has recently been detected by molecular techniques in honeybee samples collected during December in Uruguay. The presence of CBPV was apparently associated with episodes of bee mortality but without the trembling and crawling symptoms typical of paralysis outbreaks.^22^ Infectivity tests and laboratory experiments to investigate the incidence and prevalence of CBPV in Britain indicated that the virus was endemic in many apparently healthy colonies with no regular seasonal cycle of occurrence.^23^

The first molecular detection of CBPV in Iran reported by Moharrami and Modirrousta.^24^ They reported 7.50% of Iranian apiaries infected with CBPV. 

The current research is the first study of phylogenetic analysis of honeybee viruses in Iran. In the present study, we evaluated the molecular presence of CBPV in the bee samples collected from sick colonies of suspected 89 Iranian apiaries were conducted. Bee samples were sent to the laboratory for diagnosis of viral infections in affected colonies. This study is a part of honeybee viral infections assessment program in Iran, where there has been an unusual loss in adult bee population and significant honeybee mortality during the year. Out of the 89 apiaries examined, 3 (3.37%) were infected with CBPV. 

The present study looked into the first phylogenetic analysis of CBPV in suspected bee colonies and identified remarkable differences in the distribution pattern of the viruses in the different geographic regions of Iran.

A total number of 89 honeybee samples originating from four provinces of Iran, were investigated by RT-PCR for the molecular detection of CBPV. The RT-PCR assays were detected CBPV RNAs. Out of the 89 apiaries examined, 3 (3.37%) were infected with CBPV (Khorasan Razavi: 2, Kurdistan: 0, Hormozgan: 0, Mazandaran: 1). The most abundance of CBPV was in Khorasan Razavi. In contrast, the samples of Kurdistan and Hormozgan were lacking CBPV. In total, 3 RT-PCR amplification products were sequenced in both directions. Nucleotide sequences were aligned and their phylogenetic trees were drawn The sequences were identified as CBPV sequences by a BLAST search (National Center for Biotechnology Information, National Institutes of Health, Bethesda, USA). Phylogenetic tree displayed the molecular relationship between the viruses of Iranian geographic regions and references strains. According to the phylogenetic tree (Fig. 2), Iranian CBPV isolates fell into two groups: 

Group 1: IR-CPV-GMG-1, IR-CPV-GMG-2, IR-CPV-GMG-4, and IR-CPV-GMG-6 was similar to AT34 (accession number: FJ345307), Japan-2 (accession number: AB682796), B-441 (accession number: EU122231), LN-SY (accession number: KU950353), and 23 (accession number: FJ345309) from Austria, Japan, France, China, and Belgium respectively.

Group 2: IR-CPV-GMG-3, IR-CPV-GMG-5, and IR-CPV-GMG-7 were in one group and they had the most similarities to each other. These Iranian isolates were not similar to other references strains.

According to Table 3, Iranian CBPV isolates in Group 1 (IR-CPV-GMG-2, IR-CPV-GMG-4, and IR-CPV-GMG-6) showed the most similarities to European and East Asian CBPVs (90.04% to 99.64%), however, IR-CPV-GMG-1 showed 45.18% to 59.36% similarity to these strains. Also, IR-CPV-GMG-1 showed the least similarities to other Iranian isolates in Group 1. In Group 2 isolates did not show any similarity to other CBPVs and they formed a separate group in the phylogenetic tree. 

This study identified remarkable differences in the distribution pattern of the viruses in the different geographic regions of Iran. We identified CBPV genome from different geographic regions of Iran. Trade and exchange of infected animals, contaminated equipment, and bee products between apiaries, regions, or even countries may be of greater importance in the spread of viruses. Maybe this was the reason of similarities between Iranian isolates in Group 1 and European and East Asian CBPVs. Therefore, virological investigations should be considered before import and export of bees and bee products. Further researches are needed to study the other aspects of this virus-like genetic characteristics and pathogenesis in Iran.
